# Facebook use intensity and depressive symptoms: a moderated mediation model of problematic Facebook use, age, neuroticism, and extraversion

**DOI:** 10.1186/s40359-022-00990-7

**Published:** 2022-11-28

**Authors:** Nino Gugushvili, Karin Täht, Robert A. C. Ruiter, Philippe Verduyn

**Affiliations:** 1 Department of Individual and Social Psychology, University of Tartu, Tartu, Estonia; 2 Faculty of Psychology and Neuroscience, Maastricht University, Maastricht, Netherlands

**Keywords:** Facebook use intensity, Problematic Facebook use, Depressive symptoms, Age, Neuroticism, Extraversion, Moderated mediation

## Abstract

**Background:**

Research on the relationship between Facebook use intensity and depressive symptoms has resulted in mixed findings. In contrast, problematic Facebook use has been found to be a robust predictor of depressive symptoms. This suggests that when intense Facebook use results in a problematic usage pattern, it may indirectly predict depressive symptoms. However, this mediation pathway has never been examined. Moreover, it remains unclear whether the possible indirect relationship between Facebook use intensity and depressive symptoms through problematic Facebook use is moderated by demographic (age), and personality (neuroticism and extraversion) characteristics.

**Methods:**

To address these gaps, we conducted an online cross-sectional study (*n *= 210, 55% female, age range: 18–70 years old, M_age_ = 30.26, SD = 12.25). We measured Facebook use intensity (Facebook Intensity Scale), problematic Facebook use (Bergen Facebook Addiction Scale), depressive symptoms (Center for Epidemiologic Studies Depression Scale Revised), and neuroticism and extraversion (Ten Item Personality Inventory).

**Results:**

A mediation analysis revealed that problematic Facebook use fully mediates the relationship between Facebook use intensity and depressive symptoms. Moreover, a moderated mediation analysis demonstrated that this indirect relationship is especially strong among young users and users scoring high on neuroticism.

**Conclusions:**

These findings expand our understanding of the mechanisms underlying the relationship between Facebook use intensity and depressive symptoms and describe user characteristics that act as vulnerability factors in this relationship.

## Background

Facebook is the most often used social networking site worldwide with almost 3 billion users [1]. Many of these users engage in intense Facebook use by (a) spending a lot of time on Facebook, (b) having a substantial number of friends on Facebook, and (c) feeling emotionally connected to Facebook [2–4]. There is a public concern that intense Facebook use may negatively impact mental health and even contributed to the recent increase in depression rates observed in society [5, 6].

A large number of studies have been conducted to examine whether this concern is justified. Some studies have focused on the intensity of Facebook usage but these studies yielded mixed evidence [7, 8]. For instance, while it has been found that the intensity of Facebook use positively predicts depressive symptoms [9, 10], other studies could not replicate this effect [11, 12]. Recent meta-analytical evidence reveals a positive association between the intensity of using social networking sites and depressive symptoms but this association is small [13].

Other studies have taken a more fine-grained approach and decomposed social networking sites usage into active and passive usage types [14–17]. Active usage encompasses activities that foster interactions with other users and is assumed to enhance mental health. Passive usage pertains to content consumption without direct communication with other users and is assumed to undermine mental health [18]. While initial studies were largely consistent with this active–passive model of social networking sites use [19], recent studies resulted in mixed findings [20], illustrating that this model should be extended [21].

Several psychological mechanisms have been proposed to explain the effect of Facebook use on depressive symptoms but one key mechanism is problematic Facebook use. Notably, a large volume of studies confirms that the intensity of Facebook use is a consistent predictor of problematic Facebook use [11, 22–24], whereas findings for the relationship between active and passive use of social networking sites and problematic usage of social networking sites are mixed [25–27].

Problematic Facebook use pertains to addictive properties of Facebook use, such as an inability to cut down on one’s time spent on Facebook or using Facebook to manage one’s mood [28]. Whereas prior research on the impact of Facebook use intensity on depression is rather mixed, problematic Facebook use has been found to be a robust predictor of depressive symptoms [29]. As such, when intense Facebook use gradually develops into problematic Facebook use [22, 30], it may result in depressive symptoms. Surprisingly, however, this mediation pathway has never been tested. Moreover, the strength of this pathway may differ across individuals but user characteristics moderating this mediation pathway have not been studied.

The present study seeks to address these gaps by testing a moderated mediation model, in which the relationship between the intensity of Facebook use and depressive symptoms is mediated by problematic Facebook use, and this indirect association is moderated by demographic (age), and personality characteristics (neuroticism and extraversion). Testing this model enhances our understanding of the mechanisms underlying the relationship between Facebook use intensity and depressive symptoms and elucidates which user populations are especially vulnerable to negative consequences of intense Facebook use. It is notable that we focused on Facebook as the social networking site under study as Facebook is still the social networking platform with most users worldwide [31]. Furthermore, since past studies suggest that women are more prone to use social networking sites excessively [32, 33] and score higher on depressive symptoms [34, 35], we added gender as a control variable in our analyses.

Below, we first describe prior research on problematic Facebook use and discuss how it may act as a mediator in the relationship between the intensity of Facebook use and depressive symptoms. Next, we describe prior research on age, neuroticism and extraversion, and discuss how these user characteristics may act as vulnerability factors. At the end of the introduction, we specify our hypotheses.

### The mediating role of problematic Facebook use

Problematic Facebook use is a subtype of problematic social media use that specifically focuses on addiction-like behaviours occurring on Facebook [36, 37]. Problematic Facebook use is most often assessed via the Bergen Facebook Addiction Scale [28], which measures six components that are typical for substance addictions but then in the context of Facebook use: tolerance, withdrawal, conflict, salience, relapse, and mood modification [28, 38, 39]. The prevalence of problematic Facebook use poses a serious public health issue. This is reflected by a recent meta-analysis that summarized studies across 32 nations and found that 5 to 25% (depending on cut-off criteria) of users experience problematic Facebook use [40].

There is an ongoing debate among scholars [28, 41, 42] whether problematic use of Facebook and other types of digital technologies represent genuine behavioural addictions [43, 44]. This is reflected by some researchers preferring the term “Facebook addiction” [28, 45] or “Facebook use disorder” [46], while other researchers prefer terms such as “excessive Facebook use” [30], Facebook intrusion” [47], or “problematic Facebook use” [48, 49] to describe the same phenomenon. Considering that Facebook addiction is not (yet) officially recognized as a formal psychiatric disorder, we will follow the approach suggested by Panova and Carbonel [42] and use the term “problematic Facebook use”.

Intense use of Facebook may result in problematic Facebook use. Specifically, findings from two systematic reviews consistently showed that Facebook usage positively predicts problematic Facebook use [7, 22]. Moreover, seeking positive reinforcement (e.g., likes) [50] and entertainment usage of social networking sites [51] are positively associated with problematic use of social networking sites [52].

Why does intense Facebook use sometimes transform into an addiction-like usage pattern? The Online Social Regulation Theory (SOS-T) [53] states that people use social networking sites for self-regulation. It is assumed that different needs and goals (e.g., need for comparison, need for belongingness, and need for self-presentation) underlie usage of social networking sites. Fulfillment of these goals is strived for to achieve broader desired outcomes, such as increasing happiness or self-esteem. However, the SOS-T also argues that self-regulation on social networking sites does not necessarily lead to these desired end-states and can also be dysfunctional*.*

Additionally, Montag and colleagues [54, 55] argue that due to the Data Business Model (DBM), social networking sites, including Facebook, are designed to make people spend long periods of time on these platforms. For instance, the possibility to endlessly scroll on Facebook might lead to a state of flow and distorted time perception [56]. Experience of flow pertains to being fully immersed into an activity [57] and has been shown to be associated with problematic use of social networking sites [58]. Moreover, a personalized “newsfeed” on Facebook that displays relevant content tailored to each individual, might further encourage users to spend excessive amounts of time on Facebook. Finally, positive reinforcement derived from Facebook in the form of receiving [59] or providing [60] “likes” and “loves” activates the reward system in the brain, which is known to contribute to the maintenance of excessive usage patterns [61]. In line with this reasoning, a longitudinal study investigating the directionality between use of social networking sites and problematic use of social networking sites has found that increases in the intensity of use of social networking sites predicted problematic use of social networking sites one year later [11].

Lastly, according to the Interaction of Person-Affect-Cognition-Execution (I-PACE) model [62], when developing problematic and addiction-like Facebook usage patterns, one’s control over Facebook use declines and users experience negative consequences such as increases in negative affect [63], health-related issues, relational problems, and declined mental health [64]. Consistently, empirical studies found that problematic Facebook use is associated with negative outcomes including insomnia [65], stress [66], relationship dissatisfaction [67], anxiety [65], social anxiety [68, 69], and depressive symptoms [65, 69, 70].

Surprisingly, there are only two studies [71, 72] that directly investigated whether problematic use of social networking sites mediates the relationship between usage of these platforms and negative outcomes. Specifically, WeChat addiction was found to fully mediate the negative relationship between the intensity of WeChat use and academic performance [72] and social network site addiction was found to partially mediate the negative relationship between Instagram use and subjective well-being [71]. However, neither of these studies examined these relationships in the context of Facebook use.

### The moderating role of age, neuroticism, and extraversion

Problematic Facebook use may mediate the relationship between the intensity of Facebook use and depressive symptoms but the strength of this mediation pathway might vary across people. Specifically, not all people are equally vulnerable towards developing problematic Facebook use when engaging in intense Facebook use. According to the SOS-T [53], individual differences impact self-regulatory goals and outcomes associated with these goals.

Regarding demographic features, being young may act as a vulnerability factor in developing problematic Facebook use. Young people already had first access to digital technologies at a very young age [73] and use social networking sites for construing their identity [74], developing a sense of belonging [75], and for comparison with others [76]. Moreover, the prefrontal cortex is only fully developed at the age of 24 [77] and incomplete development of this brain region is expressed in risky [78] and addictive behaviours [78, 79]. In the context of social networking sites, it has been found that being young predicts higher levels of problematic Facebook use [28, 64, 80, 81] but the possible moderating impact of age in the relationship between the intensity of Facebook use and depressive symptoms through problematic Facebook use remains untested.

Among the core personality traits, neuroticism and extraversion have been found to be most strongly and consistently associated with use of social networking sites [82] and social networking sites addiction [83, 84]. Neuroticism pertains to frequent experiences of negative affect, moodiness, lack of emotional stability, anger, worry, frustration, and proneness to anxiety [85, 86]. Due to these features, users scoring high on neuroticism favour online communication as a less threatening alternative to face-to-face communication [87]. As such, they use social networking sites for strategic self-presentations [88] and compensation for lack of real-life social support [89]. In turn, the gratification of these social needs places neurotic users at a greater risk of developing an addiction-like dependency on social networking sites [83, 90]. Furthermore, according to the vulnerability model of neuroticism [91, 92], individuals with high levels of neuroticism are more vulnerable to develop addiction-like behaviour due to negative biases in attention and interpretation, and usage of maladaptive coping strategies [92]. Consistently, neuroticism has been found to have a significant positive relationship with different kinds of problematic usage of technologies [91] but the possible moderating impact of neuroticism in the relationship between the intensity of Facebook use and depressive symptoms through problematic Facebook use remains untested.

People scoring high on extraversion are warm, assertive, gregarious, highly active, impulsive, and experience often positive emotions [85]. Moreover, extraverted individuals are reward-seeking and highly sociable, and Facebook provides ample opportunities for social interaction and active self-presentation. It has been shown that on social networking sites, extraverted individuals fulfil their needs for self-presentation [93], mood enhancement (e.g., maximization of positive affect), and social needs (e.g., connection and communication) [90]. Moreover, it has been found that extraverted individuals tend to have larger online social networks, post more status updates, and photos, engage more frequently in social activities and receive more positive feedback (e.g., likes) than introverted users [89]. In turn, positive feedback [83], and pleasurable emotions [90] derived from use of social networking sites may be associated with problematic use of social networking sites for extraverts. Consistently, prior empirical research reveals that a higher level of extraversion is positively related with problematic use of social networking sites [28, 68, 94, 95]. Furthermore, among the different types of digital technology addictions, only social networking sites addiction is associated with extraversion [84]. Nevertheless, it remains unknown whether extraversion moderates the relationship between the intensity of Facebook use and depressive symptoms through problematic Facebook use.

### The present study

**Fig. 1:**
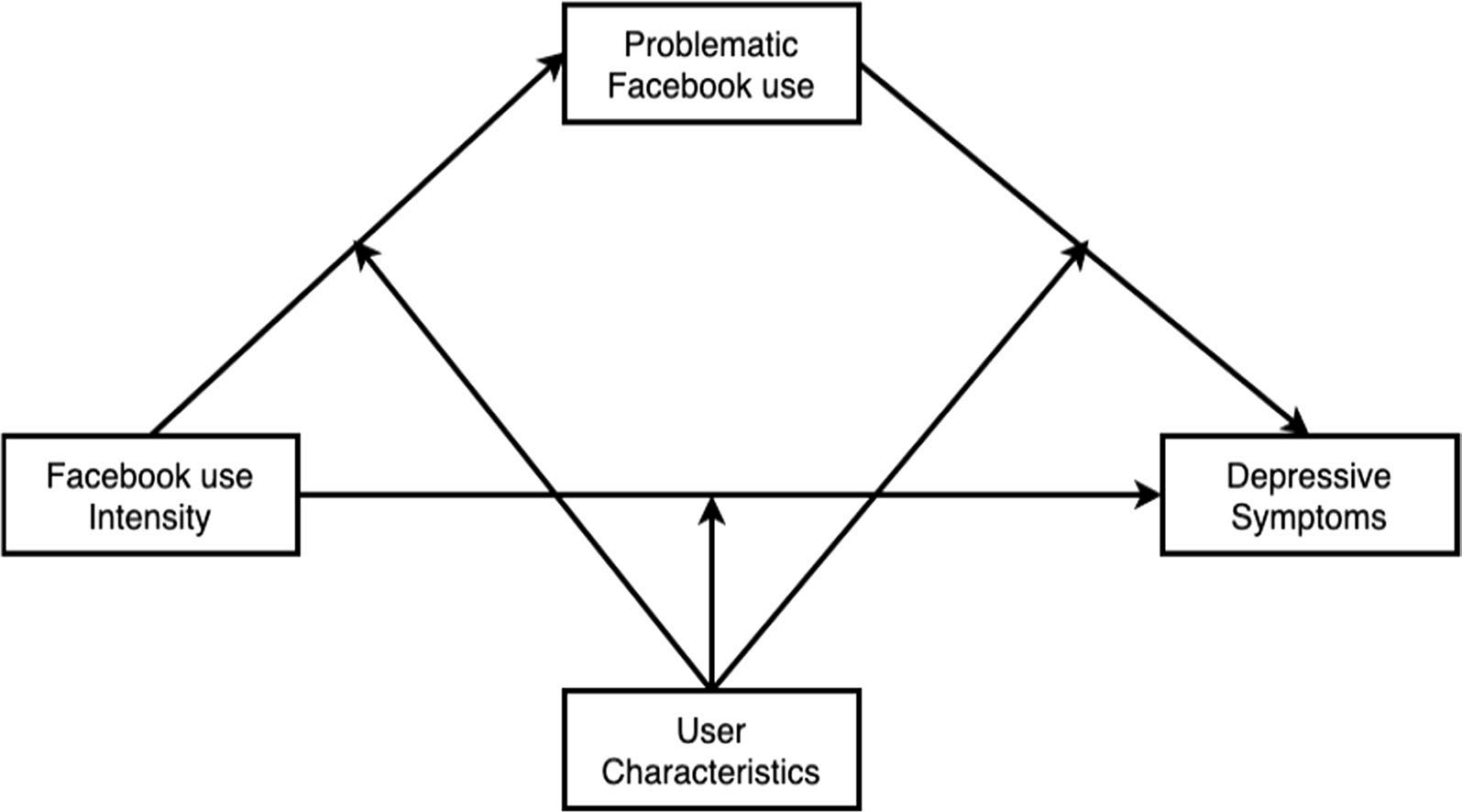
Conceptual model of the relationship between the intensity of Facebook use and depressive symptoms mediated by problematic Facebook use and moderated by user characteristics (age, neuroticism, and extraversion)

The aim of the current study is to contribute to our understanding of the relationship between the intensity of Facebook use and depressive symptoms by examining the possible mediating role of problematic Facebook use and moderating role of age, neuroticism, and extraversion. Specifically, we will test four models. First, we will test the mediating effect of problematic Facebook use in the relationship between the intensity of Facebook use and depressive symptoms. Next, we will test whether this indirect effect is moderated by age, neuroticism, and extraversion in three separate models (Fig. 1). In each moderated mediation model, we will test moderation of the relationship between the intensity of Facebook use and problematic Facebook use (path a), problematic Facebook use and depressive symptoms (path b), and the intensity of Facebook use and depressive symptoms (path c’). We have the following hypotheses:

#### H1

*The intensity of Facebook use is positively related to depressive symptoms.* Recent meta-analytical evidence reveals that the intensity of use of social networking sites (including Facebook) has a small but statistically significant positive association with depressive symptoms [13].

#### H2

*Problematic Facebook use mediates the relationship between the intensity of Facebook use and depressive symptoms.* This mediation pathway has never been directly tested but studies conducted on Instagram [71] and WeChat [72] suggest this hypothesis to hold true.

#### H3

*Age moderates the indirect relationship between the intensity of Facebook use and depressive symptoms with the relationship being stronger for younger participants.* This hypothesis has never been directly tested but it is consistent with prior research revealing a direct relation between being young and high levels of problematic Facebook use [28, 64, 80, 81].

#### H4

*Neuroticism moderates the indirect relationship between the intensity of Facebook use and depressive symptoms with the relationship being stronger for users scoring high on neuroticism.* This hypothesis has never been directly tested but it is consistent with prior evidence revealing a direct relationship between neuroticism and problematic Facebook use [91].

#### H5

*Extraversion moderates the indirect relationship between the intensity of Facebook use and depressive symptoms with the relationship being stronger for users scoring high on extraversion.* We expect this based on previous findings which suggest that the relationship between extraversion and problematic use of social networking sites is positive and significant [28, 68, 83, 90, 94, 95].

## Methods

### Participants

Participants were recruited using a convenience sampling approach. The online questionnaire was distributed via universities’ mailing list and Facebook. To take part in the study, participants had to be at least 18 years old, have a Facebook account, and provide informed consent. The initial sample consisted of 228 individuals who volunteered to participate and provided informed consent, but 12 participants did not provide information regarding their age, and six participants were younger than 18. The final sample therefore consisted of 210 participants (55% female and 45% male) with an age range from 18 to 70 (M_age_ = 30.26, SD = 12.25). Overall, the questionnaire completion rate was very high (Facebook use intensity: 98%; Problematic Facebook use: 97%; Depressive symptoms: 96%; Age: 100%; Neuroticism: 93%; Extraversion: 93%). Most participants had obtained a bachelor’s degree (42%), followed by high school degree (30.1%), master’s degree (18.2%), trade/technical/vocational training (6.7%) and doctoral degree (2.4%). Furthermore, about half of the sample were students (53.1%) while 40.7% indicated that they were employed, 3.3% reported they were retired, and 2.9% were unemployed.^1^

### Procedure & materials

Upon providing informed consent, participants answered a number of demographic questions and completed a set of questionnaires. The study took place online and was approved by the Ethics Review Committee of Maastricht University.

### Intensity of Facebook use

Facebook usage intensity was measured with the Facebook Intensity Scale [2]. It consists of eight items in total. The first six items are attitudinal questions regarding one’s emotional investment and connection with Facebook. Example items include “Facebook is part of my everyday activity” and “I feel I am part of the Facebook community”. These items were rated on a five-point Likert scale, ranging from 1 (strongly disagree) to 5 (strongly agree). The final two items assess the total number of friends one has on Facebook and the average amount of time one has spent on Facebook daily in the past week. The total number of friends is rated on an ordinal scale ranging from 1 (10 or less friends) to 9 (more than 400 friends) and the amount of time spent on Facebook is rated on an ordinal scale ranging from 1 (10 min or less) to 6 (more than 3 h). Before calculating a mean score across the eight items for each participant, all items were standardized because different items were measured on different scales. Higher scores on this scale indicate higher intensity of Facebook usage. Cronbach’s alpha for the Facebook intensity scale in the present study is 0.82.

### Problematic Facebook use

To measure problematic Facebook use we utilized the Bergen Facebook Addiction Scale [28]. This scale contains six items and measures the core aspects of addiction: tolerance, withdrawal, conflict, salience, relapse, and mood modification. These items are rated on a five-point Likert scale, from 1 (very rarely) to 5 (very often). For example, participants are instructed to answer how often during the last year they have “spent a lot of time thinking about Facebook or planned use of Facebook?” and “Used Facebook so much that it has had a negative impact on your job/studies?” We computed the mean score across the six items for each participant. Higher scores on this scale reflect higher problematic Facebook use. Cronbach’s alpha for this measure in the present study is 0.85.

### Depressive symptoms

We measured depressive symptoms by the Center for Epidemiologic Studies Depression Scale Revised (CESD-R) [96]. This scale contains 20 items, such as “I felt sad” and “I felt like a bad person”. All items were rated on a scale from 0 (Not at all or less than 1 day) to 4 (Nearly every day for 2 weeks). Respondents are instructed to answer how often they felt this way during the past two weeks. The scale score was calculated by averaging the 20 items for each participant. Higher scores on this scale indicate higher levels of depressive symptoms. Cronbach’s alpha for this scale in the present study is 0.94.

### Neuroticism

Neuroticism was assessed by the two-item neuroticism subscale from the Ten Item Personality Inventory [97]. Participants were instructed to answer the extent to which the following traits applied to them: “Anxious, easily upset”, “Calm, emotionally stable”. Both items were rated on a seven-point Likert scale ranging from 1 (disagree strongly) to 7 (agree strongly). The second item was reverse-coded such that higher scores reflect higher levels of neuroticism. As both items were correlated (r = 0.52), we calculated the mean across both items.

### Extraversion

Extraversion was assessed by the extraversion subscale from the Ten Item personality Inventory [97]. This subscale consists of two items: “Extraverted, enthusiastic” and “Reserved, quiet”. Respondents were instructed to answer the extent to which these traits apply to them. Both items were rated on a seven-point Likert scale ranging from 1 (disagree strongly) to 7 (agree strongly). The second item was reverse coded such that higher scores reflect higher levels of extraversion. As both items were correlated (r = 0.39), we calculated the mean across both items.

### Statistical approach

We used IBM SPSS (version 27) for data analysis. After computing descriptive statistics and bivariate correlations among the key variables, we made use of the Process Macro (version 3.5.3) [98] to examine our main hypotheses. First, using model 4, we checked whether problematic Facebook use mediated the relationship between the intensity of Facebook use and depressive symptoms. To test the moderating effect of user characteristics, we fitted three separate models in the SPSS PROCESS macro, specifically model 59 [98] with age, neuroticism, or extraversion as moderator. According to Hayes [98], conditional indirect effects (moderation) are established if either path a (i.e., the relationship between the intensity of Facebook use and problematic Facebook use) or path b (i.e., the relationship between problematic Facebook use and depressive symptoms) or both are influenced by the moderating variable. In all models, continuous predictors were centered and a bootstrapping procedure across 10,000 samples was utilized. Moreover, in all models, we controlled for the effects of gender on problematic use of Facebook and depressive symptoms. Gender was coded in the following way: 0 denotes males, 1 denotes females. Please note that we used the bootstrapping technique, therefore, it was not necessary to meet the assumptions with regard to the mediation model outlined by Hayes [98]. Furthermore, the variance Inflation Factors (VIF) were all below 5, indicating the absence of multicollinearity [99].

## Results

Table 1 displays the descriptive statistics and bivariate correlations among the assessed variables.

**Table 1:** Descriptive statistics and correlations between intensity of Facebook use (standardized), problematic Facebook use, depressive symptoms, neuroticism, extraversion, and age

	Mean	SD	2	3	4	5	6
1. Facebook use intensity	0	0.69	.593**	.221**	.158*	.014	− .122
2. Problematic Facebook use	1.88	0.79		.454**	.320**	− .151*	− .139*
3. Depressive symptoms	0.66	0.65			.521**	− .177*	− .302**
4. Neuroticism	3.61	1.60				− .151*	− .153*
5. Extraversion	4.41	1.56					.027
6. Age	30.26	12.25					

^*^p < .05, ^**^p < .01

### Does the intensity of Facebook use predict depressive symptoms?

To answers this question, we conducted a regression analysis predicting depressive symptoms by Facebook use intensity. The intensity of Facebook use was found to be positively related to depressive symptoms (*B* = 0.191, β = 0.211, *SE* = 0.063, *p* = 0.003).

### Does problematic Facebook use mediate the relationship between the intensity of Facebook use and depressive symptoms?

**Fig. 2:**
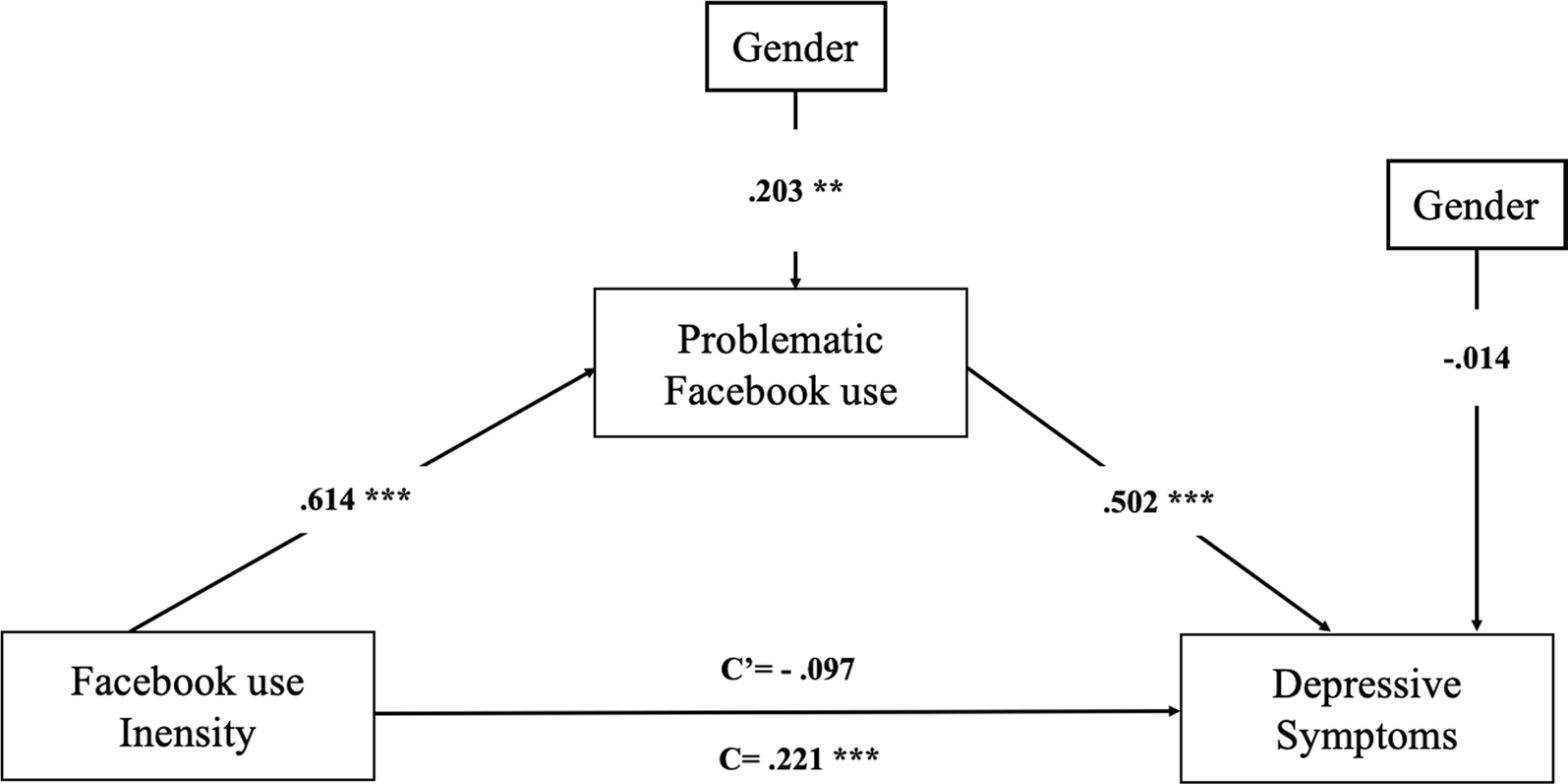
Problematic Facebook use as a significant mediator of the relationship between the intensity of Facebook use and depressive symptoms (Controlling for Gender). *Note N* = 200. Regression weights are standardized. C’ is the direct effect of Facebook use intensity on Depressive symptoms; C is the total effect of Facebook use intensity on Depressive symptoms. ^***^p < .01

The results showed that the overall model predicting problematic Facebook use was significant: *F*(2, 197) = 63.56, *p* < 0.001, R^2^ = 0.392. Intensity of Facebook use significantly and positively predicted problematic Facebook use (*B* = 0.706, β = 0.614, *SE* = 0.064, *p* < 0.001). The overall model predicting depressive symptoms was also significant: *F*(3, 196) = 16.47, *p* < 0.001, R^2^ = 0.201. Problematic Facebook use significantly and positively predicted depressive symptoms (*B* = 0.396, β = 0.502, *SE* = 0.065, *p* < 0.001). The indirect effect of the intensity of Facebook use on depressive symptoms through problematic Facebook use was statistically significant (*B* = 0.279, β = 0.308, *SE* = 0.056, Bootstrap 95% CI: [0.175—0.396]). Finally, the direct association between the intensity of Facebook use on depressive symptoms was not significant (*B* = -0.088, β = -0.097, *SE* = 0.074, *p* = 0.235) reflecting full mediation (see Fig. 2).

#### The moderating role of age

**Fig. 3:**
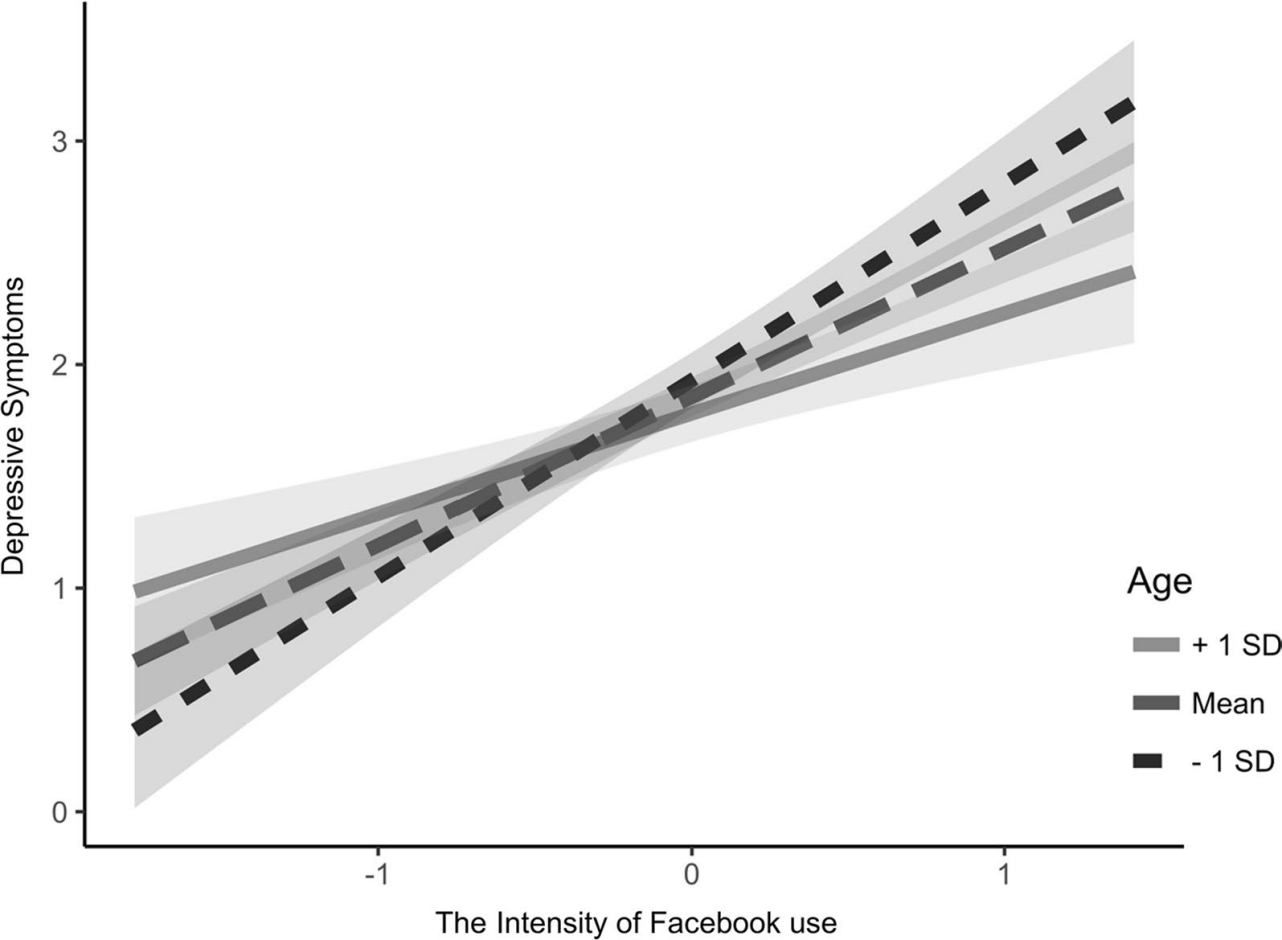
Problematic Facebook use as a function of Facebook use intensity for people scoring 1SD below and 1SD above the mean age

The overall model predicting problematic Facebook use was significant F (4, 195) = 37.06, *p* < 0.001. We found that the interaction term between the intensity of Facebook use and age significantly predicted problematic Facebook use (Table 2). To facilitate the interpretation of this moderation effect, Fig. 3 displays problematic Facebook use as a function of the intensity of Facebook use and age (1SD below the mean, 1 SD above the mean). Results from the simple slope tests indicate that the association between the intensity of Facebook use and problematic Facebook use is significant for both age groups. However, the relationship between the intensity of Facebook use and problematic Facebook use is stronger among younger users *(B* = 0.902, *SE* = 0.088, *p* < 0.001*)*, compared to older users *(B* = 0.464, *SE* = 0.093, *p* < 0.001*)*.

**Table 2:** The moderating role of age in the indirect relationship between the intensity of Facebook use and depressive symptoms through problematic Facebook use

	Problematic Facebook use			Depressive symptoms		
	*b*	*SE*	*p*	*b*	*SE*	*p*
Gender	.343	.087	< .001	.009	.083	.915
Facebook use intensity	.683	.063	< .001	− .107	.073	.143
Age	− .007	.004	.038	− .013	.003	< .001
Problematic Facebook use				.365	.065	< .001
Facebook use intensity × Age	− .018	.005	.001	.002	.005	.745
Problematic Facebook use × Age				− .004	.005	.424
R^2^	.432			.269		

*N* = 200

On the other hand, although the overall model predicting depressive symptoms was significant *F*(6, 193) = 11.83, *p* < 0.001, neither the interaction term between Facebook use intensity and age, nor between problematic Facebook use and age were significant (Table 2). Overall, age was found to moderate the indirect relationship between the intensity of Facebook use and depressive symptoms by moderating the relationship between the intensity of Facebook use and problematic Facebook use (path a). Consistently, the indirect relationship between the intensity of Facebook use and depressive symptoms through problematic Facebook use was moderated by age. The indirect effect was more pronounced among younger users (*B* = 0.377, *SE* = 0.087, Bootstrap 95% CI: [0.208, 0.553]), compared to older users (*B* = 0.145, *SE* = 0.057, Bootstrap 95% CI: [0.061, 0.285]).

#### The moderating role of neuroticism

**Fig. 4:**
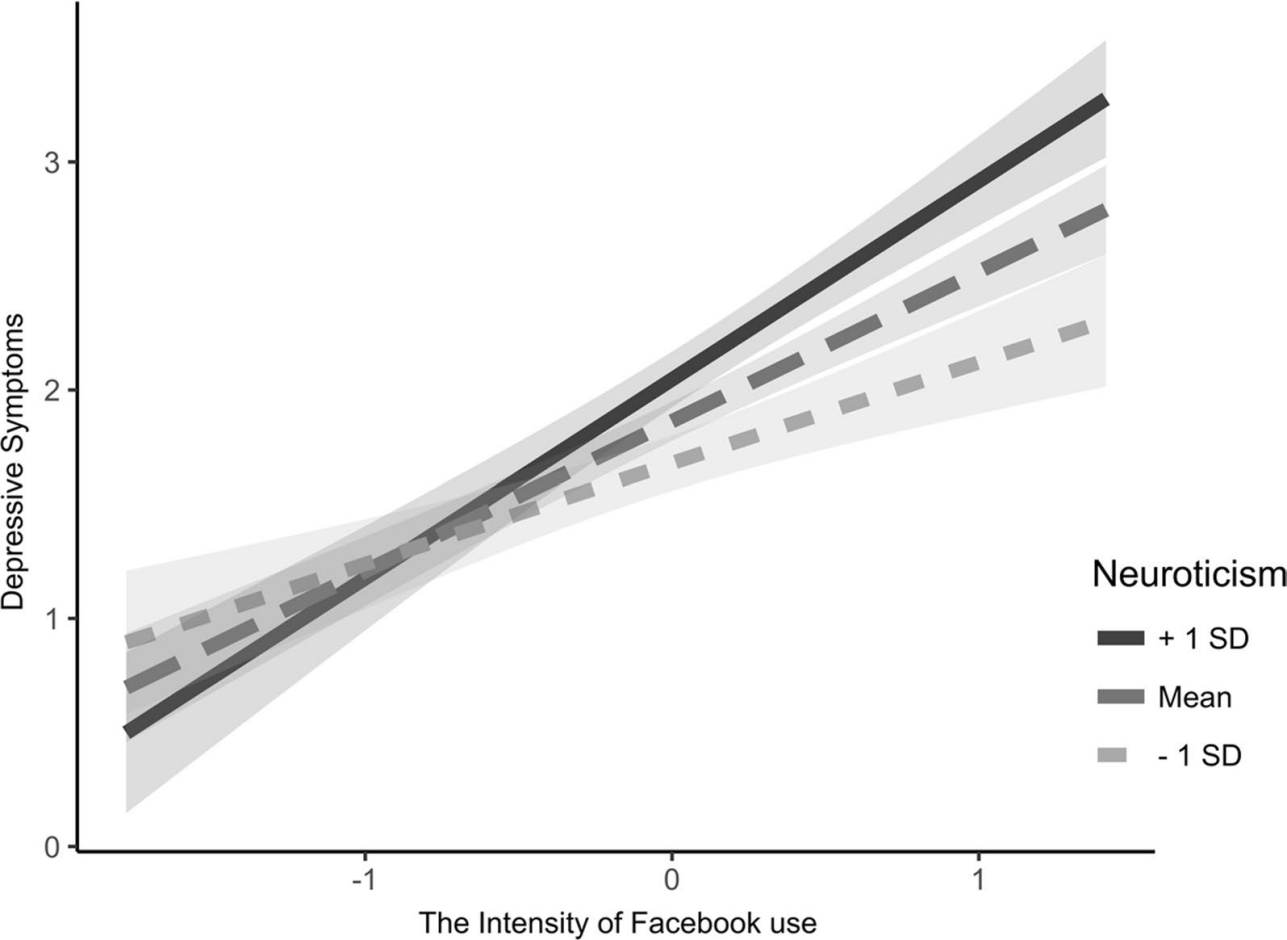
Problematic Facebook use as a function of Facebook use intensity for people scoring 1SD below and 1SD above the mean score of neuroticism

Next, we examined the possible moderating effect of neuroticism (Table 3). The overall model predicting problematic Facebook use was significant: *F*(4, 189) = 41.92, *p* < 0.001. The relationship between the intensity of Facebook use and problematic Facebook use was moderated by neuroticism (*B* = 0.140, *SE* = 0.038, *p* < 0.001). To ease the interpretation of this interaction effect, Fig. 4 shows the predicted value of problematic Facebook use as a function of the intensity of Facebook use and neuroticism (1SD below the mean, 1SD above the mean). Simple slopes analysis revealed that the intensity of Facebook use is related to problematic Facebook use at both levels of neuroticism, but this relationship is considerably weaker for users scoring low on neuroticism (*B* = 0.447, *SE* = 0.087, *p* < 0.001), compared to users scoring high on neuroticism *(B* = 0.902, *SE* = 0.088, *p* < 0.001).

**Table 3:** The moderating role of neuroticism in the indirect relationship between the intensity of Facebook use and depressive symptoms through problematic Facebook use

	Problematic Facebook use			Depressive symptoms		
	*b*	*SE*	*p*	*b*	*SE*	*p*
Gender	.286	.086	.001	− .024	.077	.757
Facebook use Intensity	.679	.063	< .001	− .053	.070	.449
Neuroticism	.106	.027	< .001	.162	.025	< .001
Problematic Facebook use				.273	.065	< .001
Facebook use Intensity × Neuroticism	.140	.038	< .001	− .003	.043	.948
Problematic Facebook use × Neuroticism				.020	.038	.586
R^2^	.470			.353		

*N* = 194

Furthermore, the overall model predicting depressive symptoms was significant as well: *F*(6, 187) = 17.01, *p* < 0.001. However, the interactions between Facebook use intensity and neuroticism and between problematic Facebook use and neuroticism were not significant (Table 3).

Overall, neuroticism was found to moderate the indirect relationship between the intensity of Facebook use and depressive symptoms by moderating the relationship between the intensity of Facebook use and problematic Facebook use (path a). Consistently, the indirect relationship between the intensity of Facebook use and depressive symptoms through problematic Facebook use was moderated by neuroticism. The indirect effect was more pronounced among users scoring high on neuroticism (*B* = 0.275, *SE* = 0.097, Bootstrap 95% CI: [0.094, 0.476]), compared to users scoring low on neuroticism (*B* = 0.110, *SE* = 0.044, Bootstrap 95% CI: [0.036, 0.209]).

#### The moderating role of extraversion

Finally, we tested the possible moderating effect of extraversion (Table 4). The overall model predicting problematic Facebook use was significant *F*(4, 189) = 33.69, *p* < 0.001. However, the interaction term between the intensity of Facebook use and extraversion when predicting problematic Facebook use was not significant (Table 4). Similarly, while the overall model predicting depressive symptoms was significant: *F*(6, 187) = 8.49, *p* < 0.001, the interaction terms between Facebook use intensity and extraversion and between problematic Facebook use and extraversion were not significant (Table 4). Overall, extraversion was not found to moderate the indirect relationship between the intensity of Facebook use and depressive symptoms.

**Table 4:** The moderating role of extraversion in the indirect relationship between the intensity of Facebook use and depressive symptoms through problematic Facebook use

	Problematic Facebook use			Depressive symptoms		
	*b*	*SE*	*p*	*b*	*SE*	*p*
Gender	.283	.091	.002	− .001	.086	.995
Facebook use Intensity	.717	.065	< .001	− .054	.077	.486
Extraversion	− .074	.029	.010	− .036	.027	.178
Problematic Facebook use				.373	.067	< .001
Facebook use Intensity × Extraversion	− .051	.041	.216	− .008	.049	.866
Problematic Facebook use × Extraversion				.020	.046	.654
R^2^	.416			.214		

*N* = 194

### Sensitivity analysis

In the preceding analyses, we examined the role of each moderator separately. To demonstrate the robustness of our findings, we reran the aforementioned three moderation analyses controlling for the other two moderators. For example, we re-examined the moderating role of age, while controlling for the effects of neuroticism and extraversion. These sensitivity analyses confirmed our conclusions: age and neuroticism moderate the relationship between the intensity of Facebook use and problematic Facebook use, whereas extraversion has no moderating effect.

## Discussion

The aim of our study was to examine the relationship between the intensity of Facebook use and depressive symptoms and study the mediating and moderating mechanisms underlying and affecting this relationship. We found that problematic Facebook use fully mediated the relationship between the intensity of Facebook use and depressive symptoms. Moreover, we demonstrated that age and neuroticism moderated the first stage of this mediation relationship (path a: intensity of Facebook use predicting problematic Facebook use) but not the second stage (path b: problematic Facebook use predicting depressive symptoms). Extraversion did not moderate the indirect relationship between the intensity of Facebook use and depressive symptoms. We discuss these findings in more detail below.

### The mediating role of problematic Facebook use

Our first aim was to investigate whether problematic Facebook use underlies the relationship between the intensity of Facebook usage and depressive symptoms. Our results confirmed that problematic Facebook use fully mediates this relationship. This finding is line with prior research showing that intensive Facebook usage can transform into problematic usage patterns [65, 100, 101], which in turn, can lead to declines in mental health [45, 48, 70]. Moreover, our results are consistent with previous studies directly examining the mediating roles of social networking site addiction and WeChat addiction in the relationship between Instagram use and subjective well-being [71] and WeChat use and academic performance [72], respectively. However, our study extends these findings by suggesting that also on Facebook intense but non-addictive use of this social network site may gradually transform into an addictive usage pattern.

Why does problematic Facebook use mediate the relationship between intense Facebook use and depressive symptoms? According to the Online Self-Regulation Theory [53], users resort to using social networking sites to regulate themselves. However, this attempt is not always successful and can result in detrimental outcomes. As such, it is plausible that the specific goals and motivations that underlie self-regulatory use of social networking sites seem achievable to users and make them more invested in Facebook usage. This, in turn, can lead to an overreliance on Facebook and the development of problematic usage of Facebook.

Additionally, Montag and colleagues [54] argue that the Data Business Model (DBM) also plays a role in the development of problematic usage of social networking sites. Specifically, it is assumed that the medium-specific factors play an important role in the development of problematic usage behaviours. Therefore, it seems plausible that specific elements and parts of the design of Facebook make people more vulnerable to develop problematic usage patterns. For instance, the experience of flow [102] and distorted perception of time while using Facebook may contribute to problematic usage patterns [103]. Moreover, once addiction-like attachment towards digital technology (including Facebook) is being developed, users may tend to lose control over their behaviour over time which results in negative outcomes for mental health [62].

### The moderating roles of age and neuroticism but not extraversion

The second aim of the present study was to test whether the indirect relationship between the intensity of Facebook use and depressive symptoms via problematic Facebook use was conditional on age, neuroticism, and extraversion. With regard to age, we found that being older acts as a protective factor as the relationship between the intensity of Facebook use and problematic Facebook use is less strong compared to younger users. Interestingly, we were unable to find a moderating impact of age on the second stage of the mediation pathway connecting problematic Facebook use to depressive symptoms. As such, this suggests that age only plays a protective role at the onset of developing addiction-like problematic behaviours on Facebook but does not protect the user from developing depressive symptoms once the user is already engaging in problematic Facebook use.

Our results corroborate previous studies showing that younger users have heightened risks to develop technology addictions [104, 105] including problematic Facebook use [28, 64]. Our findings also support the SOS-T model [53], which maintains that individual differences influence self-regulatory goals on social networking sites. As such, our finding specify for whom self-regulation on social networking sites may become dysfunctional. A number of reasons may explain the observed relationships. First, compared to older adults, young people may engage in more social comparisons on Facebook which may results in more intense Facebook use for reasons of impression management [76]. Second, it has been shown that young users who experience a higher need to belong are at greater risk of developing problematic usage patterns [106]. Alternatively, the present finding suggesting that young users are especially vulnerable towards developing problematic Facebook use may also be rooted in incomplete development of the prefrontal cortex which makes individuals vulnerable towards developing addiction-like symptoms [78, 107, 108].

We also found that neuroticism is a vulnerability factor as it moderates the indirect relationship between the intensity of Facebook use and depressive symptoms. Specifically, we demonstrated that higher levels of neuroticism amplify the relationship between the intensity of Facebook use and problematic Facebook use. Similar to age, neuroticism did not moderate the relationship between problematic Facebook use and depressive symptoms.

While previous research has consistently linked higher levels of neuroticism with different types of digital addictions [91, 109], our study has taken this research one step further by showing that neuroticism not only directly predicts problematic Facebook use, but also interacts with Facebook usage intensity in fostering problematic Facebook usage. Overall, our findings fit well into the vulnerability model of neuroticism [91, 92] which argues that higher levels of neuroticism presents an important risk factor for developing common mental health disorders. Moreover, our results complement the SOS-T model [53] which posits that especially users with emotion regulation difficulties are prone to resort to dysfunctional self-regulation via social networking sites [16]. Here, we again clarify the specific subset of users for whom self-regulation on social networking sites is associated with detriments. As such, while neurotic users may turn to these platforms to regulate their need for self-presentation [88] or satisfy their need to belong [93], they may fail doing in an efficient manner resulting in problematic social networking sites use. This is consistent with research showing a negative relationship between neuroticism and emotion regulation capacity [110].

Finally, we were unable to find evidence for a moderating impact of extraversion on the indirect relationship between the intensity of Facebook use and depressive symptoms. Previous research on the relationship between extraversion and digital technologies suggested a positive link between problematic Facebook use and extraversion [28, 68, 94, 95]. However, our results suggest that extraversion is neither a vulnerability nor a protective factor in the indirect relationship between the intensity of Facebook use and depressive symptoms through problematic Facebook use. Given, that extraverts tend to have larger offline social networks [111], are satisfied with their offline social relationships [112], and use healthy coping strategies [113, 114], it is plausible to argue that they satisfy their needs for self-presentation and rewarding experiences both online and offline. As a result, extraverts are not dependent on social networking sites such as Facebook to regulate themselves and are not more prone to develop addictive patterns following intense Facebook use.

### Implications and limitations

Our findings are useful for counsellors, public health policy makers, and researchers. We have demonstrated that (non-problematic) intense Facebook use may result in problematic Facebook usage patterns, which, in turn, undermine users’ mental health. Moreover, we have shown that this indirect effect is especially outspoken among young users and users with high levels of neuroticism. Counsellors can make use of these findings to better identify and support the most vulnerable user populations. Moreover, they could rely on our findings when designing interventions, as the present study suggest that intense Facebook use is especially detrimental for mental health when it results in addictive usage patterns. Similarly, public health policy makers could make use of our findings when creating prevention strategies and campaigns by warning especially young users and users high in neuroticism of the possible dangers of excessive Facebook use and associated addiction symptoms. Moreover, also researchers who examine the relationship between usage of social networking sites and mental health might benefit from our findings. Specifically, our study directly responds to a call made by Boer and colleagues [11] to identify groups of users for whom usage of social networking sites develops into problematic usage patterns. However, future studies are needed to further examine problematic Facebook use as a key mechanism underlying the relationship between Facebook use intensity and mental health, and to pay more attention to individual differences that either protect or make users more vulnerable towards negative outcomes of intense Facebook use. Additionally, future studies should study a broad range of different social networking sites and their unique features when examining the relationship between use of social networking sites and problematic usage patterns. The present study provides evidence that intensive Facebook use may turn into problematic usage. Moreover, this was also found for Instagram [71] and WeChat [72] in prior studies, but more evidence is necessary to identify the platforms for which usage is most likely to turn into problematic usage patterns.

The present study has also a number of limitations that should be taken into account when interpreting the findings of this study. First, we used a convenience sampling technique and Facebook users with higher education degrees were overrepresented. Future studies would benefit from recruiting bigger and more diverse samples. Second, while the sample size of the present study was modest, we ran a sensitivity analysis to check the range of effect sizes (two-tailed correlation) that our study design could detect reliably (80% power), given our sample size (n = 210) and α = 0.05. The results show that our study design is sufficiently powered to detect effect sizes from |*p*| 0.19 and above, i.e., small to medium-sized effects (and larger) as specified by Cohen [115]. This is consistent with the effect sizes observed in previous research on the relationship between the constructs under examination [48, 116, 117]. However, future studies using larger sample sizes are necessary to replicate the current findings. Such larger sample sizes would also allow modelling the data with a structural equation modelling approach rather than the PROCESS approach used in the present study. Third, in the present study, we focused on Facebook. While Facebook still has the most users worldwide [118], other platforms such as TikTok and Instagram are becoming increasingly popular, especially among younger users [31]. Future studies are needed to examine whether our findings replicate across these other platforms, and to test whether our findings also hold when assessing social networking sites use in general without specifying specific platforms. Fourth, future studies need to replicate our findings by using different conceptualizations or subtypes of social networking sites use (e.g., active and passive usage) which may be differentially related to mental health outcomes [19] and problematic social networking site usage [26]. Fifth, in this study we made use of the Ten Item Personality Inventory (TIPI) [97]. While the neuroticism and extraversion dimensions of the TIPI have been shown to have good convergence with the corresponding dimensions of the International Personality Item Pool Neo-120 and Big Five Inventory-2 [119], future research needs to replicate our findings using longer measures of extraversion and neuroticism. Furthermore, our study was cross-sectional. As such, strong causal claims cannot be made. While we examined the mediating role of problematic Facebook use in the relationship between Facebook use intensity and depressive symptoms, alternative models, including bi-directional relationships between these constructs are also possible [120]. Future studies using longitudinal or experimental designs are needed to clarify the temporal and causal relationships between the intensity of Facebook use, problematic Facebook use and depressive symptoms. Finally, future research using clinical samples is needed to advance our understanding of social networking sites usage in a clinical context.

## Conclusions

In the present study, we have demonstrated that the relationship between the intensity of Facebook use and depressive symptoms is mediated by problematic Facebook use. Moreover, we have shown that this indirect relationship is moderated by age and neuroticism. Overall, our results suggest that intense Facebook use can result in adverse outcomes once it turns into a problematic (addictive) usage pattern, which is especially likely to happen among users who are young and score high on neuroticism.

## Data Availability

The dataset used and analyzed during the current study are available from the corresponding author on reasonable request. Moreover, upon publication, the dataset will be made accessible on dataverseNL.

## Abbreviations

The I-Pace model: The Interaction of Person-Affect-Cognition-Execution (I-PACE) model
SPSS: Statistical product and service solutions

## Footnotes

1 The dataset along the code will be accessible on dataverseNL.
